# Genome-Wide Binding of MBD2 Reveals Strong Preference for Highly Methylated Loci

**DOI:** 10.1371/journal.pone.0099603

**Published:** 2014-06-13

**Authors:** Roberta Menafra, Arie B. Brinkman, Filomena Matarese, Gianluigi Franci, Stefanie J. J. Bartels, Luan Nguyen, Takashi Shimbo, Paul A. Wade, Nina C. Hubner, Hendrik G. Stunnenberg

**Affiliations:** 1 Department of Molecular Biology, Nijmegen Centre for Molecular Life Sciences, Radboud University Nijmegen, Nijmegen, The Netherlands; 2 Laboratory of Molecular Carcinogenesis, National Institute of Environmental Health Sciences, Research Triangle Park, North Carolina, United States of America; Institute of Genetics and Molecular and Cellular Biology, France

## Abstract

MBD2 is a subunit of the NuRD complex that is postulated to mediate gene repression via recruitment of the complex to methylated DNA. In this study we adopted an MBD2 tagging-approach to study its genome wide binding characteristics. We show that in vivo MBD2 is mainly recruited to CpG island promoters that are highly methylated. Interestingly, MBD2 binds around 1 kb downstream of the transcription start site of a subset of ∼400 CpG island promoters that are characterized by the presence of active histone marks, RNA polymerase II (Pol2) and low to medium gene expression levels and H3K36me3 deposition. These tagged-MBD2 binding sites in MCF-7 show increased methylation in a cohort of primary breast cancers but not in normal breast samples, suggesting a putative role for MBD2 in breast cancer.

## Introduction

DNA methylation is the covalent addition of a methyl group to the cytosine of a CpG dinucleotide and represents an important epigenetic mechanism involved in several biological processes like X-inactivation [1], differentiation [2], genomic imprinting [3] and cancer [4].

Around 70% of the CpG dinucleotides (CpGs) in the mammalian genome are methylated [5] except for CpG islands (CGIs), regions of high CpG density, that are usually unmethylated [6]. However, CGIs promoters are not always unmethylated and can acquire methylation during differentiation [7] or they can be aberrantly methylated in cancer [8]. Methylation of promoter CpG-islands is correlated to transcriptional repression [9] whereas recent evidences show genome-body methylation being mainly associated with transcriptional activity [10], [11] [12].

It has been proposed that one of the mechanisms of transcriptional repression mediated by CpGs methylation involves binding of methyl-CpG-binding proteins (MBPs) to the methylated cytosine and recruitment of a co-repressor complex [13]. MBPs are divided in three different families: MBD (methyl binding domain), Kaiso and SRA domain proteins [14]. The MBD family includes MeCP2, MBD1, MBD2 and MBD4 that can bind methylated DNA via the Methyl-CpG Binding Domain (MBD) while three other members of this family namely MBD3, MBD5 and MBD6 do not bind methylated DNA [15].

MBD2 is a subunit of the Mi2-NuRD complex that was previously shown to mediate gene repression via recruitment of the complex to methylated promoters [16], [17]. Since promoter hyper-methylation is a well-known hallmark of cancer, several studies linked MBD2 function to epigenetic regulation of genes critical during carcinogenesis [18], [19], however, most of these studies looked at a limited number of target genes. Recent studies challenged the model of MBD2 as a predominantly promoter-proximal repressor suggesting that binding could also regulate activity of target genes [20].

Although it has been widely shown that MBD2 selectively binds methylated DNA in vitro [21], [22] the proof that this also occurs *in vivo* was only recently provided by genome wide binding of MBD2 and other family members by comprehensive chromatin immunoprecipitation (ChIP) sequencing [23], [24]. Genome wide mapping of MBD2 binding in mouse embryonic stem cells showed that *in vivo* binding predominantly occurs at highly methylated, CpG dense regions, although a subset of binding sites was detected at active unmethylated promoters. Another recent study in HeLa cells showed that MBD2 mainly binds promoters of inactive genes and extrapolated this observation to imply that MBD2 acts as repressor at those regions [23].

To gain further insights into the function of and epigenetic regulation by MBD2 we generated a tagged version of the protein and stably expressed it in the MCF-7 cell line. We mapped genome wide binding of MBD2 by ChIP sequencing (ChIP-seq) and together with base resolution whole genome bisulfite sequencing (WGBS) we determined the methylation content and the possible role of methylation density at MBD2 enriched regions. We also categorized the epigenetic make up of MBD2 binding sites taking advantage of a large body of ChIP-seq data and detected MBD2 at a subset of lowly active promoters. Regions bound by MBD2 in MCF-7 show overall increased methylation levels in a large set of primary breast cancer samples but not in a model of non-cancer human mammary epithelial cells.

## Results and Discussion

### Tagged MBD2 incorporates into native NuRD-complex

**Figure 1:**
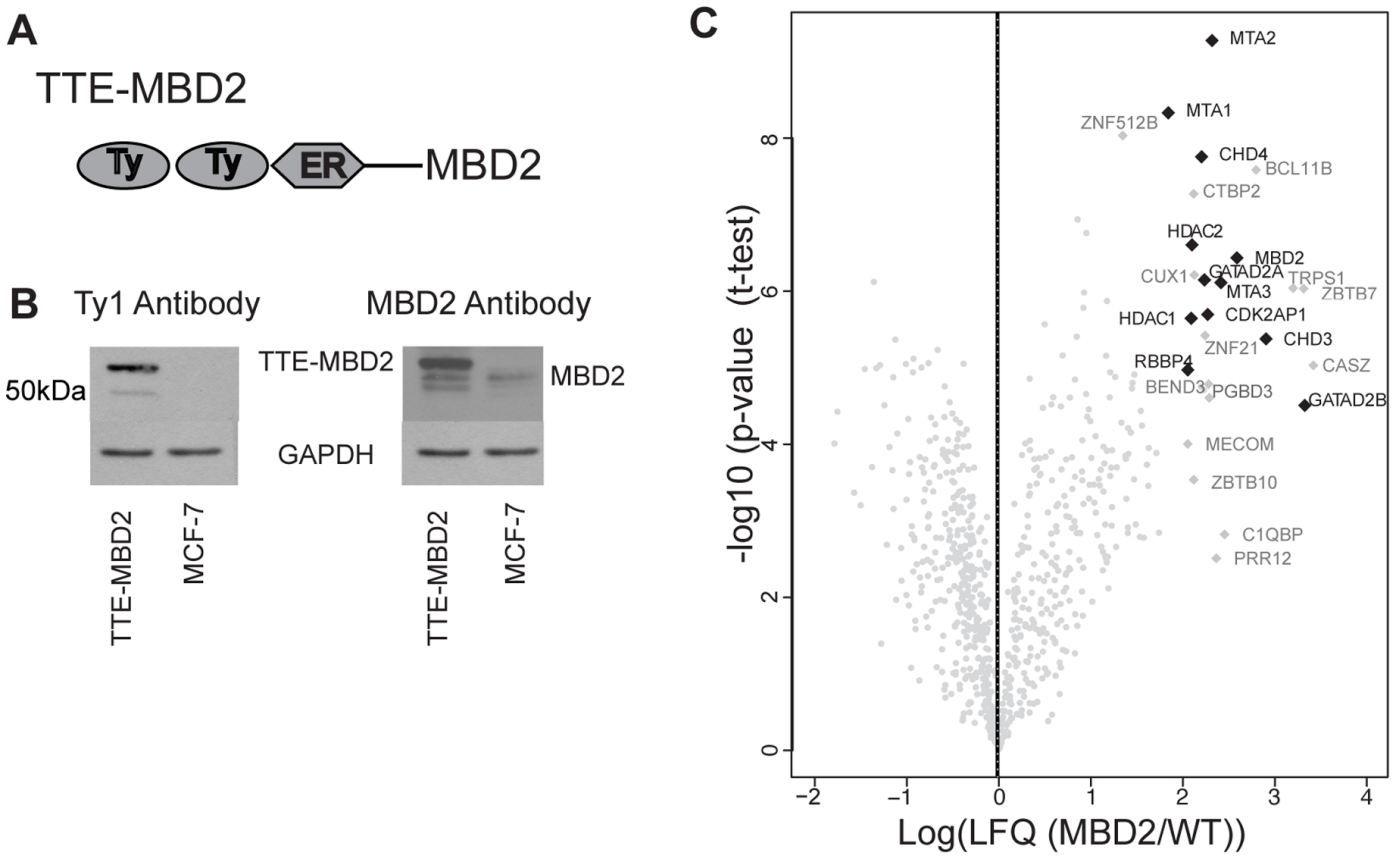
Generation of a tagged MBD2. a) Schematic presentation of tagging approach: double Ty1 and ER epitopes are inserted at the N-terminal of human full length MBD2. b) Western blot on whole cell lysates from TTE-MBD2 MCF-7 and WT MCF-7. Antibodies against tag (Ty1) and MBD2 are used. GAPDH is shown as loading control. c) Volcano plot showing results from Mass Spectrometric Analysis of immunoprecipitation experiment. The x-axis shows the log of ratios between LFQ intensities in TTE-MBD2 against the control WT. The y-axis display −log10 of the p-value calculated by a permutation-based FDR-corrected *t* test. The black dots underline Mi2-NuRD complex components within the significantly enriched interactors (grey dots).

In order to study the genome wide binding of MBD2 we generated a tagged version (hereafter referred as TTE-MBD2, Fig 1A) stably expressed in MCF-7 as at the initiation of this study, the available commercial MBD2 antibodies were of low quality and/or not suited for ChIP-seq. The epitope tag (TY1-TY1-ER) had already been successfully used for IP and ChIP applications [25] and was placed at the N-terminus of the protein. TTE-MBD2 expressed in MCF-7 cells is specifically recognized by the Ty1 antibody (Fig1B left panel), that does not detect the endogenous protein in wild type MCF-7 cells (WT). Western blot analysis with an MBD2-specific antibody showed that the tagged protein is expressed considerably higher as compared to endogenous MBD2 levels (Fig 1B, right panel).

Next we performed large-scale Ty1 immunoprecipitation on both TTE-MBD2 and WT cells followed by mass spectrometry analysis in order to determine whether the exogenous TTE-MBD2 is assembled into a NuRD complex. Results from triplicate pull-downs were analyzed with MaxQuant and label-free quantitation (LFQ) intensities were used to determine statistically enriched MBD2 interactors as previously described [26]. Amongst the 25 most significantly enriched interactors, the well-known MBD2-NuRD complex components CHD3/4, GATAD2A/B, MTA1/2/3, HDAC1/2 and CDK2AP1 (DOC-1) are co-precipitated (Fig1 C, black squares). We further detected RBBP4 but not RBAP46 (RBBP7) which share 90% sequence homology. A list with the specific interactors is reported (Table 1) and a full list of all identified proteins is also available (Table S1). Interestingly, among the specific interactors we could detect several zinc-finger and other DNA binding proteins. Our findings indicate that the tagged MBD2 is at least in part incorporated in a NuRD-like complex [21] and thus that the tag unlikely interferes with the composition of the complex.

**Table 1:** List of TTE-MBD2 specific interactors identified from Mass Spectrometry.

Gene names	Protein names	(-)Log t-test p value	t-test Difference
**MTA1**	Metastasis-associated protein MTA1	8.32954	1.84248
**ZNF512B**	Zinc finger protein 512B	8.03327	1.34558
**CHD4**	Chromodomain-helicase-DNA-binding protein 4	7.75574	2.20372
**BCL11B**	B-cell lymphoma/leukemia 11B	7.58416	2.79627
**CTBP2**	C-terminal-binding protein 2	7.27166	2.11806
**HDAC2**	Histone deacetylase 2;Histone deacetylase	6.60528	2.09939
**CUX1**	Homeobox protein cut-like 1	6.17975	2.21916
**GATAD2A**	Transcriptional repressor p66-alpha	6.14678	2.2355
**MTA3**	Metastasis-associated protein MTA3	6.1107	2.4135
**TRPS1**	Zinc finger transcription factor Trps1	6.04	3.19905
**ZBTB7B**	Zinc finger and BTB domain-containing protein 7B	6.03263	3.31228
**CDK2AP1**	Cyclin-dependent kinase 2-associated protein 1	5.69484	2.27067
**HDAC1**	Histone deacetylase 1	5.64503	2.09078
**ZNF219**	Zinc finger protein 219	5.42088	2.24232
**CHD3**	Chromodomain-helicase-DNA-binding protein 3	5.37692	2.90386
**CASZ1**	Zinc finger protein castor homolog 1	5.03132	3.4185
**RBBP4**	Histone-binding protein RBBP4	4.97025	2.05424
**BEND3**	BEN domain-containing protein 3	4.75216	2.19638
**PGBD3**	PiggyBac transposable element-derived protein 3	4.70819	2.23163
**GATAD2B**	Transcriptional repressor p66-beta	4.50829	3.32597
**MECOM**	MDS1 and EVI1 complex locus protein EVI1	4.00392	2.05347
**ZBTB10**	Zinc finger and BTB domain-containing protein 10	3.53701	2.11845
**C1QBP**	Complement component 1 Q subcomponent-binding protein	2.822	2.45405
**PRR12**	Proline-rich protein 12	2.51089	2.36333

25 most significantly enriched TTE-MBD2 interactors, identified after Ty1 immunoprecipitation on both TTE-MBD2 and WT cells followed by mass spectrometry analysis. Results from triplicate pull-downs were analyzed with MaxQuant and label-free quantitation (LFQ) intensities were used to determine statistically enriched MBD2 interactors. Immunoprecipitation from wild type (WT) MCF-7 was used as a control.

### MBD2 is preferentially associated with CGI-promoters

**Figure 2:**
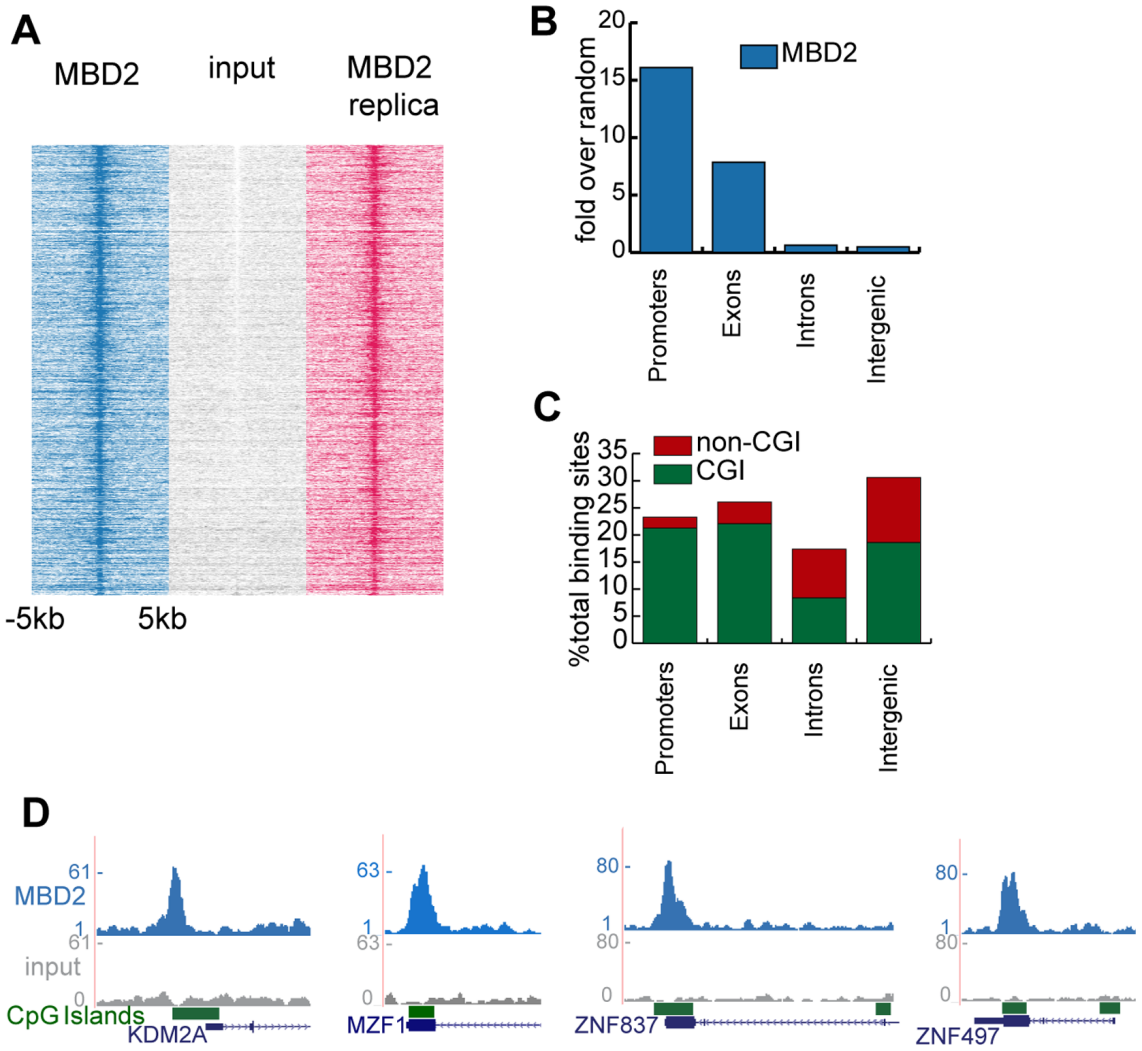
Genome-wide binding of TTE-MBD2. a) Heatmap displaying tag densities in TTE-MBD2 (left and right) and input (middle) at MBD2 binding sites around 5 kb up- and downstream of the center of the peaks. b) Genomic location of peaks: each category is expressed as fold over random (y-axis), the random set consists of an equal number of sites having on average same length of the peaks. c) CpG content of each category expressed as percentage of the total binding sites (y-axis). d) Screenshots from the genome browser showing example of CGI promoters (KDM2A) and exons (MZF1, ZNF837, ZNF497) binding.

In order to assess genome wide binding of MBD2 we isolated chromatin from TTE-MBD2 and WT MCF-7 cells, and performed ChIP sequencing. To preserve protein-protein interactions we stabilized the MBD2-NuRD complex on chromatin by applying a double crosslinking method [27]. Cells were first treated with 1.5 mM disuccinimidil glutarate (DSG) before applying the standard formaldehyde fixation. Along with Ty1-tag antibody (Fig S1), we also used antibodies against MBD2 that became commercially available in the course of the study. ChIP-seq using the Ty1 antibody on WT MCF-7 cells was performed as a negative control (data not shown) to correct for possible antibody cross-reactivity. One of the commercial MBD2 antibodies showed the highest signal over background data compared to Ty1 ChIP and was subsequently used in the data analysis. MBD2 binding sites were identified using MACS 2.0 [28] with a p-value of 10e^6^ resulting in 8349 peaks. A pile-up heatmap showing quantitation of tags 5 kb up- and downstream the center of MBD2 binding sites (Fig 2A left panel) revealed strong signal intensity, which is absent in the input (Fig 2A middle panel). A biological replica of a MBD2 ChIP-seq experiment revealed high reproducibility of the data (Fig 2A right panel). Specificity of the binding was validated by ChIP-qPCR on TTE-MBD2 and wild type MCF-7 cells using the MBD2 as well as Ty1 antibody (Fig S1). The MBD2 binding sites with highest tag density were also enriched in Ty1 ChIP-seq using TTE-MBD2 but not in WT MCF-7 cells (Fig S2).

The genomic distribution of MBD2 binding sites was determined in comparison to a random set of genomic regions with the same average length, representative of the total genomic sequence distribution (Fig 2B). Genomic features such as intron, intergenic, exons and promoters were subsequently grouped accordingly to their GC content (Fig 2C). Binding preferentially occurs at CpG islands (CGIs) in particular at promoters and to a lower extent at exons as previously reported [24]. Representative examples of MBD2 binding are shown (Fig 2D). Strong binding is observed at the CGI promoter of KDM2 gene as well as at the CGI exons of MZF1, ZNF837 and ZNF497.

### Methylation density plays a critical role in MBD2 binding

**Figure 3:**
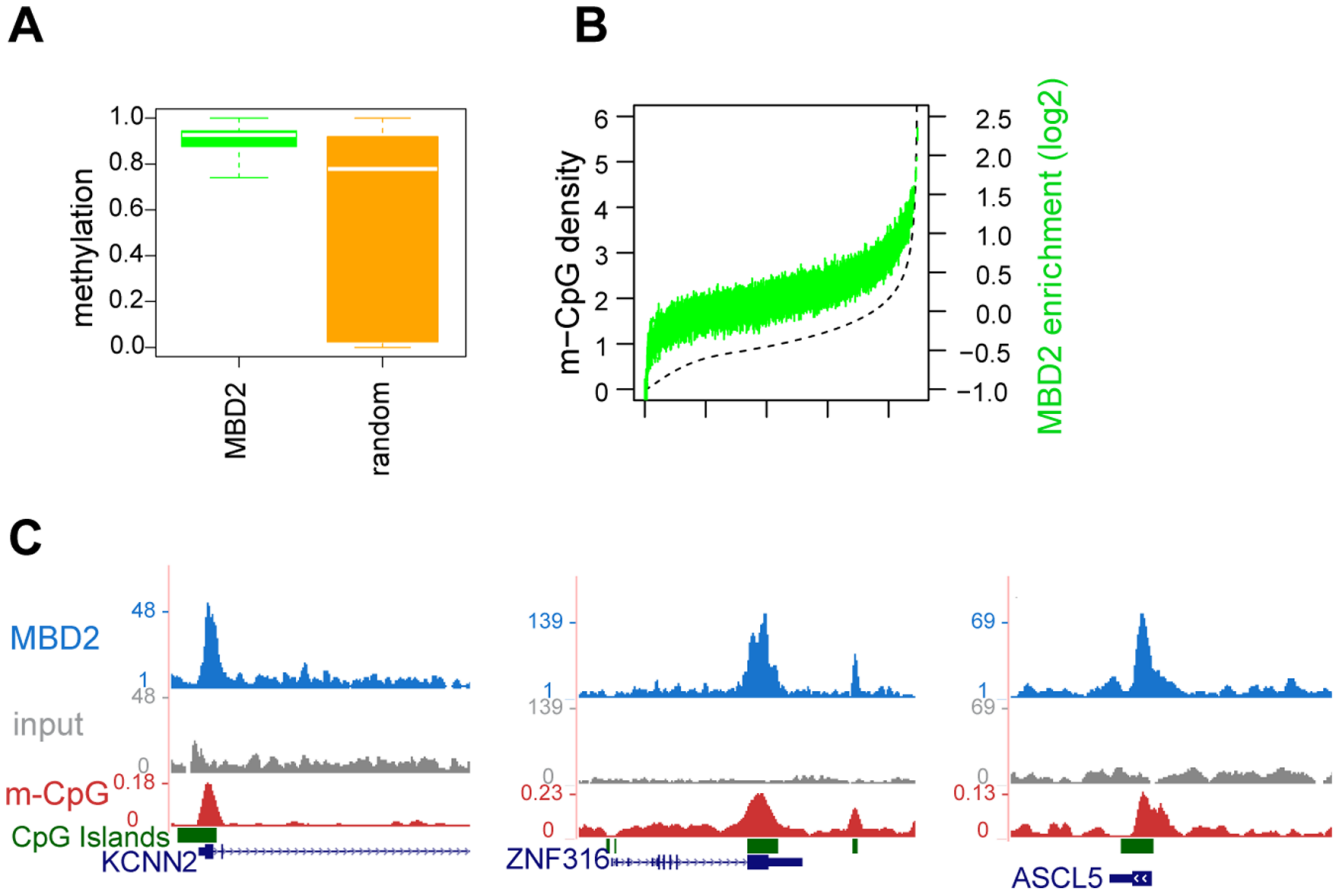
Methylation state at MBD2 binding sites. a) Boxplot displaying methylation level at TTE-MBD2 binding sites compared to random (0 = 0% methylation, 1 = 100% methylation). b) Genome wide correlation between TTE-MBD2 enrichment (green) and methylation density, calculated at 1 kb windows ranked by methylation density (dashed line). c) Screenshots from genome browser showing correlation between CpG methylation density (red track) and TTE-MBD2 peaks at KCNN2, ZNF316, and ASCL5.

A large body of data strongly suggests that MBD2 specifically binds to methylated DNA in vitro [16], [21] but evidence for its binding to methylated DNA in vivo was only recently obtained in mouse embryonic stem cells [24]. Therefore, we assessed the DNA methylation levels at MBD2 binding sites. Base pair resolution whole genome bisulfite sequencing (WGBS) was performed on MCF-7 cells as recently described [29]. Bisulfite conversion rate was around 99.9% and 51.4 mean coverage of CpG dinucleotides. The percentage of DNA methylation underneath MBD2 peaks was determined and compared with methylation levels at a random set of genomic regions matched for genomic distribution of MBD2 binding sites. MBD2 binding was clearly enriched at methylated genomic loci since more than 80% of the binding sites had DNA methylation levels between 80-100% (Fig 3A), whereas in the random set the level ranges from 10 to 90%. In order to check the correlation between methyl-CpG density and MBD2 binding genome wide we binned the genome in 1 kb windows and calculated MBD2 enrichment over input and methylation density within each window. Methylation density was expressed as the sum of methylated CpGs for each CpG dinucleotide normalized by length. Next we ranked the windows according to their methylation density (Fig 3B dashed line) and calculated MBD2 enrichment (green plot). A strong correlation between the two variables was detected as previously observed in mouse ESCs [24]. Figure 3C shows representative examples; the red track displays methyl-CpG density computed over 50 bp windows across the genome. MBD2 peaks clearly coincided with increased methylation density as also exemplified for KCNN2, ZNF316, and ASCL5.

### A subset of MBD2 binding sites marks active CpG island promoters

To further characterize MBD2 binding sites we determined the epigenetic marks at the enriched loci, to gain insight into a putative role of MBD2 in gene regulation. We performed RNA polymerase II (Pol2), H2A.Zac and H3K4me3 ChIP-seq in MCF-7 as well as strand-specific RNA-seq, while other data-sets were retrieved from the ENCODE project. Histone marks and transcription factors associated with active promoters (Pol2, H2A.Zac, H3K4me3, H3K36me3, H3K27ac) as well as enhancers (H3K27ac, P300), repressive marks (H3K27me3, H3K9me3) and methyl CpG (mCpG) levels were selected for this analysis. Note that the epigenetic profiles were generated in wild type MCF-7 cells, not in the line over-expressing TTE-MBD2.

**Figure 4:**
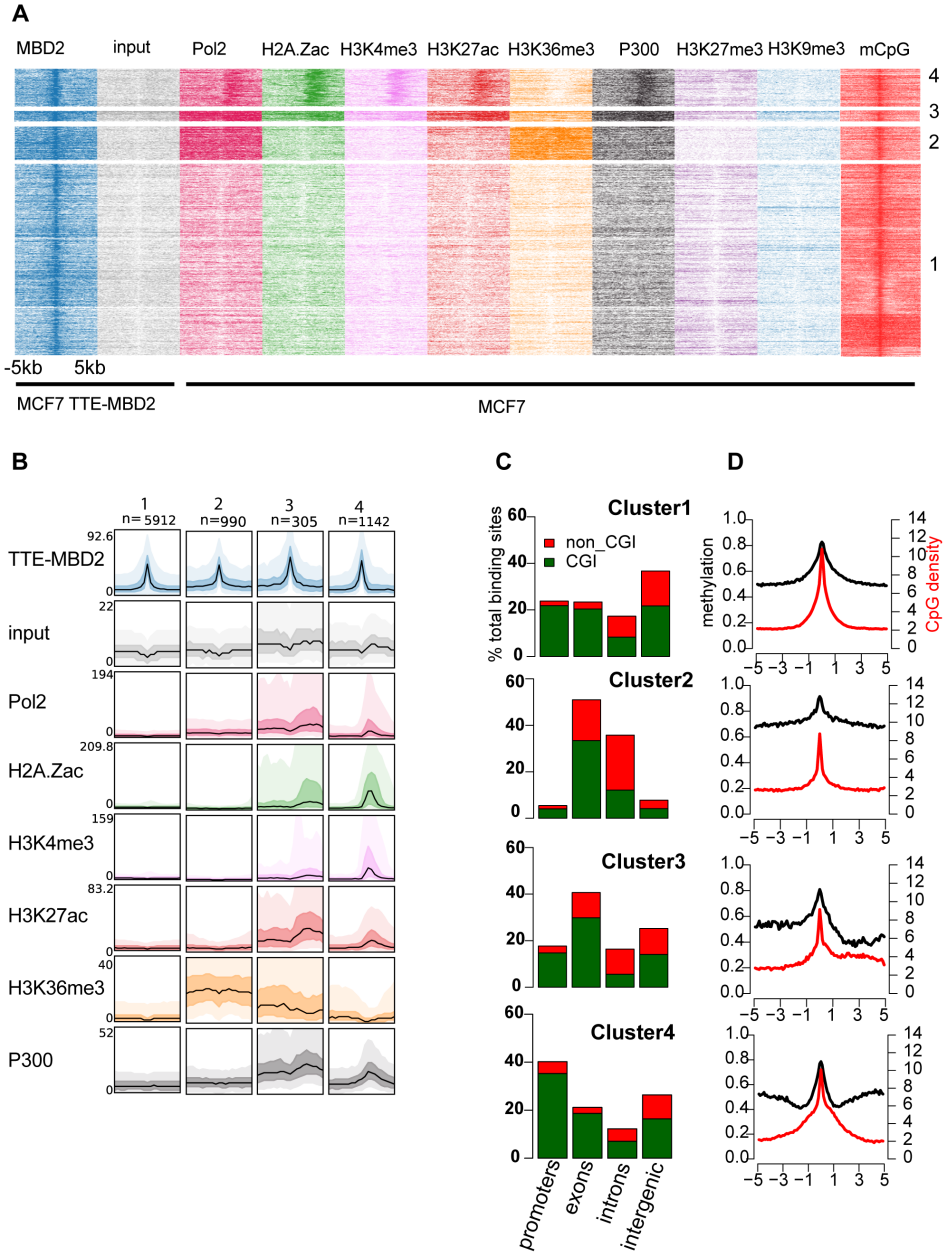
A subset of MBD2 binding sites associate with promoters enriched for active marks. a) Heatmap of signal density using k-means clustering on TTE-MBD2 peaks (5 kb up/downstream the center of MBD2 peaks) for TTE-MBD2, input, Pol2, H2A.Zac, H3K4me3, H3K27ac, H3K36me3, P300. The clustering shows four different groups of binding sites (numbers indicated on the right). H3K27me3 H3K9me3 and mCpG levels are not participating to the clustering itself but average signal density is calculated according to the clustering order. b) Average profiles for each clusters of the indicated histone marks/transcription factors 5 kb up and downstream the middle of each binding site, windows were divided in 20 bp bin. The mean enrichment is shown using a black line and the 50th and 90th percentile are also displayed using a dark and light color respectively. c) Genomic location of binding sites within each cluster was calculated as for Figure 2C. d) Average methylation (black line) and average CpG density (red line) for all binding sites in each cluster, 5 kb up- and downstream the center of the peaks in 100 bp window.

For each dataset we calculated tag densities 5 kb up and downstream of the center of the MBD2 binding sites and performed k-means clustering to group the binding sites into distinct clusters. Heatmaps showing the output of the clustering (Fig 4A) clearly depict 4 main clusters of binding sites. For this analysis we did not take into account directionality of transcription since the reference was the center of the binding sites. Two mirrored clusters were merged into cluster 4. Average profiles for each cluster (Fig 4B black lines) underpinned the distribution of the marks and their overall signal densities in each cluster. The 50^th^ and 90^th^ percentile of the distributions are also represented as a dark and light shadow respectively. Genomic distribution for each group was calculated (Fig 4C) together with average methylation levels and CpG density (Fig 4D).

The largest cluster, number 1, is comprised of MBD2 binding sites with a clear enrichment of DNA methylation and CpG density but no co-occurrence of any of the epigenetic marks analyzed. The binding sites are roughly equally distributed over CGI promoters, exons and intergenic regions (about 20% in each category). Cluster 2 comprises MBD2 binding sites in CGI located in exons and introns. Interestingly this group of loci showed high levels of H3K36me3 and DNA methylation level was high up- and downstream the binding sites, probably reflecting gene body methylation pattern [30], [31].

Cluster 3 is a small cluster slightly enriched for CGI exons. Average methylation levels showed an asymmetric pattern, probably consistent with increased methylation at one flank of the binding sites.

Interestingly, cluster 4 showed strong localized enrichment of marks associated to active transcription such as H3K4me3, Pol2, H3K27ac, H2A.Zac and low but appreciable levels of H3K36me3 as well as enrichment for P300. Interestingly, the enriched signals are positioned at one side of the MBD2 binding sites rather than coinciding with MBD2. Although cluster 4 represented a small subset of the total binding sites (1142/8349) it was interesting to find MBD2 co-occurring with active marks, in line with recent findings underlining that a fraction of MBD2 binding can mark also active promoters [23], [32]. No enrichment was detected to co-occur with the histone marks H3K27me3 and H3K9me3, which are associated with transcriptional repression. The binding sites in cluster 4 were enriched for CGIs at transcription start sites (TSS-CGI) (420/1142). CpG density and methylation levels were also increased at these binding sites.

Representative examples from each cluster are shown (Fig S3): respectively the CGI promoters of FG2 (cluster 1) and ADHFE1 (cluster 2) and the CGI exons of KLF4 (cluster 3) and KCND3 (cluster 4).

### MBD2 binding downstream of active promoters affects RNA Pol2 distribution

The specific pattern of histone marks and Pol2 binding observed in cluster 4 showed that MBD2 binds close to transcriptional start sites. Intriguingly, both the epigenetic marks and Pol2 were limited to a short and discrete genomic stretch that is not consistent with variable gene length. Our subsequent analysis excluded that we were dealing with short genes. Moreover MBD2 binding in WT MCF-7 cells was confirmed for a subset of loci, to exclude possible artifacts due to the over-expression (Fig S4).

**Figure 5:**
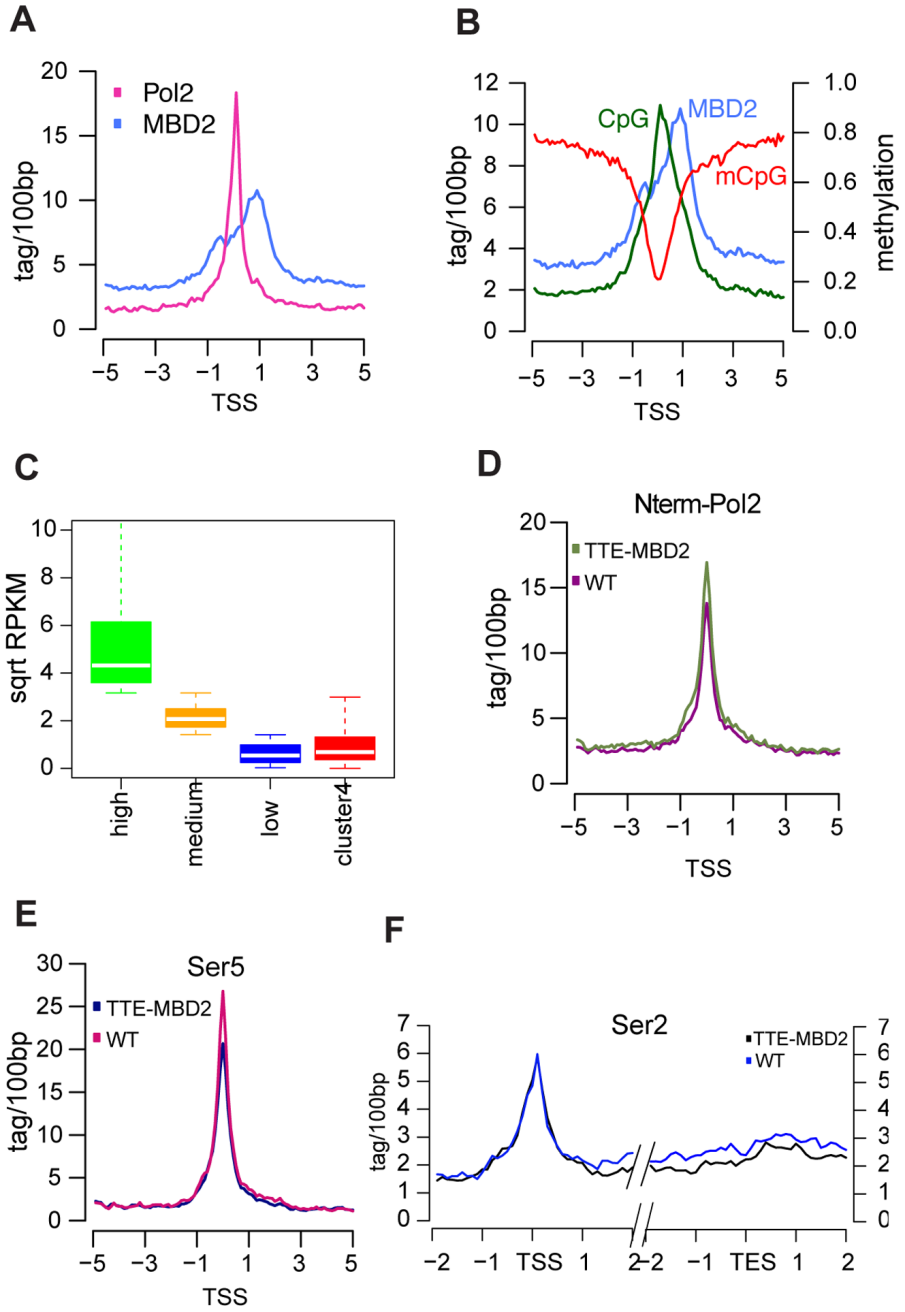
MBD2 and Pol2 distribution at active promoters from cluster 4. a) Average profile of Pol2 (MCF-7 WT) and MBD2 (TTE-MBD2) at promoters from cluster 4, calculated 5 kb up- and downstream the TSS. Average profile of CpG density, MBD2 and methylation levels at promoters from cluster 4, calculated 5 kb up- and downstream the TSS. c) Boxplots showing RPKM values for all Ref-seq annotated expressed genes sorted and divided in 3 categories according to their transcript level, compared to RPKM values for genes annotated from cluster 4. d) Average profile of N-term Pol2 at promoters from cluster 4, calculated 5 kb up- and downstream the TSS for TTE-MBD2 and WT MCF-7. e) As for 5D average profiles of Ser5 phosphorylated Pol2 at promoters from cluster 4, calculated 5 kb up- and downstream the TSS for TTE-MBD2 and WT MCF-7. f) Average profiles of Ser2 phosphorylated Pol2 over gene bodies downstream promoters from cluster 4, calculated 2 kb up- and downstream the TSS and the TES, for TTE-MBD2 and WT MCF-7.

To investigate the binding of MBD2 and its implication for transcriptional activity, we calculated the average tag density of the enriched features Pol2, and MBD2 relative to the TSS (Fig 5A) of the annotated promoters binding sites from cluster 4 (420/1142), together with CpG and methylation density (Fig 5B). At these 420 sites, MBD2 binding is located around 1 kb downstream from the TSS. These promoters are enriched for Pol2 and are CpG dense. Next, we assessed the expression levels of the genes in the same cluster. We first ranked all Ref-seq annotated genes from highest to lowest in terms of RNA transcript levels and divided them into three bins (hereafter defined as “high”, ”medium” and “low”). We then compared our subgroup of genes in cluster 4 to overall expression levels. This analysis showed that the genes in cluster 4 are indeed expressed at low to medium levels (Fig 5C). A similar analysis was performed for H3K36me3 levels, ranking genes in three categories according to their H3K36me3. This analysis confirmed that genes downstream of the promoters in cluster 4 were active and displayed H3K36me3 levels nearly reaching that of medium expressed genes (Fig S5).

Given the gene expression levels and ChIP data, MBD2 binding sites of cluster 4 may prevent full transcriptional activation i.e. poises these promoters. Alternatively, MBD2 binding downstream of the main peak might block elongation of Pol2. Therefore we performed ChIP-seq for both WT and TTE-MBD2 cells with an antibody against the N-term of Pol2 (N-20) to determine Pol2 occupancy independently of the phosphorylation status of its C-terminal domain (CTD). At the TSS from cluster 4, Pol2 occupancy was slightly higher in the TTE-MBD2 as compared to the parental MCF-7 cells (fig 5D), which was supported by increased Pol2 using the CTD antibody (Fig S6). To study whether this phenomenon is in line with pausing, we determined Pol2 distribution during initiation and elongation by performing Pol2 ChIP-seq in TTE-MBD2 and WT MCF-7 cells with antibodies specific for phosphorylation at Ser5 (initiation) and Ser2 (elongation) of the CTD [33]. If MBD2 binding affects pausing, the level of Ser5 at the promoter should be higher in TTE-MBD2 as compared to parental MCF-7 cells, instead we found the opposite (Fig 5E). Confusingly, we observed a decrease of the elongating Pol2 over gene bodies in the TTE-MBD2 that would be in line with pausing (Fig 5F). These results are consistent with the idea that MBD2 might induce a block in Pol2 elongation at these promoters.

To shed light on these seemingly contradictory results, we investigated whether the sequence composition in cluster 4 promoters and downstream regions may provide an explanation. It has recently been shown that CpG and methylation density in gene bodies has a modest negative correlation with elongation rate [34], [35]. We therefore determined mCpG density over all genes, grouped them as previously into three classes (Fig S7 “high”, “medium” and “low”) and compared them with genes in cluster 4. The CpG density of cluster 4 genes over gene bodies was in the same range as those in the high CpG bin which was supported by the DNA methylation distribution (Fig S8).

Note that in MCF-7 WT, that is in the absence of (over)expression of MBD2, this class of promoters are already decorated with active marks (Fig 4A) and transcribed at low levels (Fig 5C) which may in part be due to their sequence composition and methylation density over the gene-body. As the binding of MBD2 in TTE-MBD2 cells did not change the level of transcription (data not shown), the epigenetic and transcription state of these genes are apparently not affected by MBD2 binding.

### MBD2 binds hyper-methylated hallmarks of cancer

**Figure 6:**
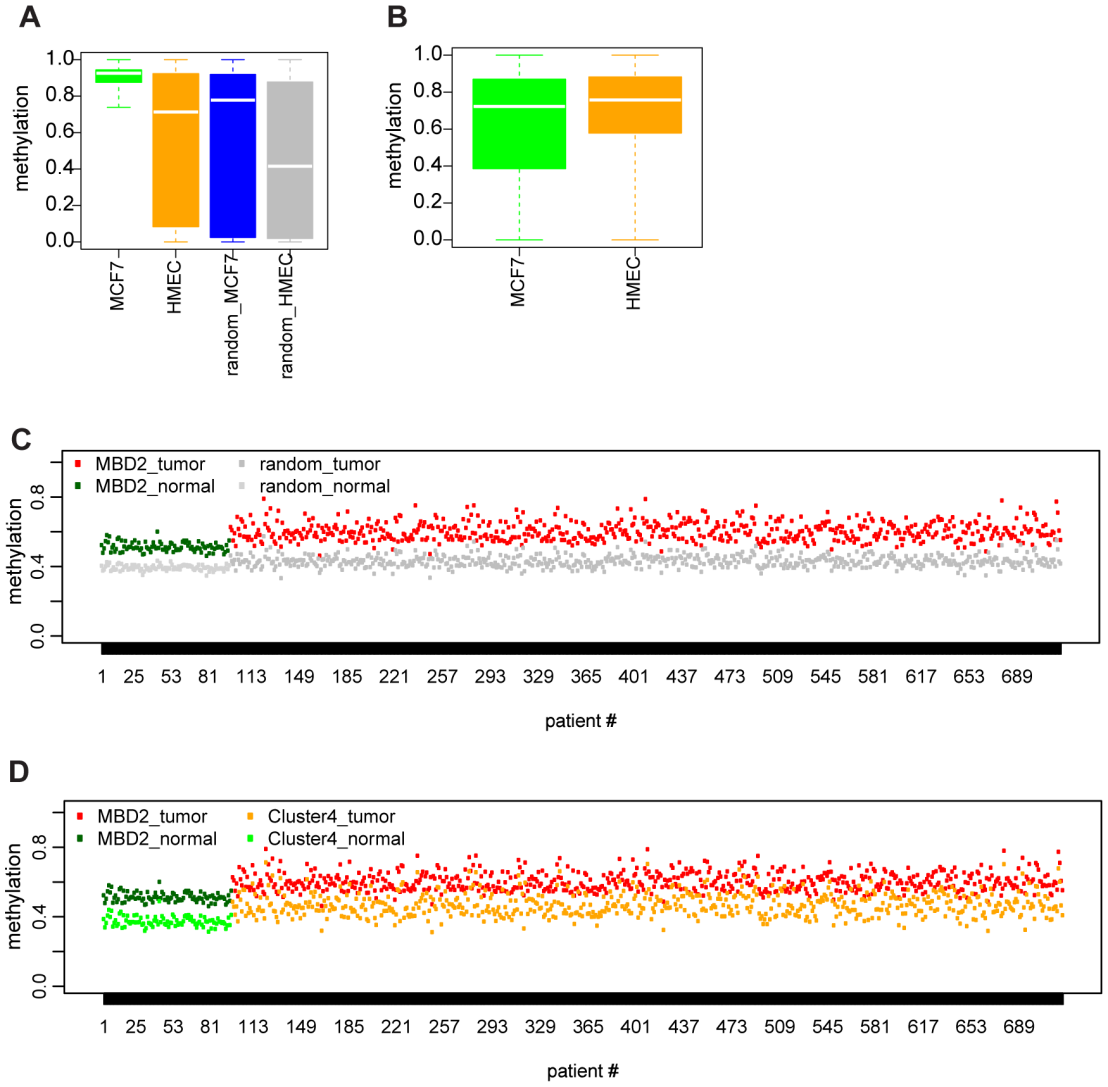
Methylation levels of MBD2 binding sites in normal and breast cancer. a) Boxplot displaying methylation levels at TTE-MBD2 binding sites in MCF-7 and HMEC compared to methylation at random regions respectively in MCF-7 and HMEC. Random is corrected for genomic distribution as for Fig 3A. b) Genome-wide methylation levels in MCF-7 and HMEC calculated in 50 bp sliding windows. c) Dot-plot showing mean methylation for each samples (#patients on X-axis) at all MBD2 binding sites: red dots are indicating mean-methylation at MBD2 sites in tumor samples (MBD2_tumor) and green for healthy samples (MBD2_normal). Same analysis at a random set of sites (as for Fig 3A) for the two datasets is depicted in grey. On the y-axis methylation levels (0 = 0% methylation, 1 = 100% methylation). d) As for Fig 6C dot-plot showing mean methylation for each samples (#patients on X-axis) at all MBD2 binding sites and at the subset represented in cluster 4.

Most of the MBD2 binding sites in the MCF-7 breast cancer cell line were located at highly methylated promoters and exonic CpG islands that are rarely methylated in normal tissues. To investigate whether the observed MBD2 binding patterns could be a feature of breast cancers, we first checked the methylation status of all MBD2 bound regions in the HMEC cell line, a model of non-cancer human mammary epithelial cells [36]. In HMEC, methylation at regions bound by MBD2 (in TTE-MBD2 MCF-7) showed a very broad methylation range from 10 to 90% similar to a random distribution (Fig 6A) even though the global methylation level in HMEC is higher than in MCF-7 (Fig 6B). The specific methylation increase at CpG rich sites MBD2 sites and the global decrease compared to normal breast tissue is in line with reported aberrations in cancer methylome: a global hypo-methylation and local hypermethylation [37].

This prompted the question whether in primary cancers hypermethylation occurred at MBD2 binding sites as in MCF-7. Therefore, we calculated the methylation status at MBD2 binding sites in a large cohort of primary breast cancers (from TCGA: breast invasive carcinoma methylation data). We computed for each cancer sample as well as for a set of samples from healthy individuals included in this TCGA study the mean methylation levels at all MBD2 binding sites. The same analysis was performed for an equal number of random sites corrected for genomic distribution (as for Fig 3A). Interestingly, most of the tumor samples showed increased mean methylation at all MBD2 binding sites as compared to normal samples (Fig 6C), whereas increased methylation was not observed at random sites.

We extended our analysis to MBD2 regions in cluster 4, comparing mean methylation levels at binding sites from this specific group between tumor and normal to mean methylation at all MBD2 sites (Fig 6D). Also in this case we observed a statistically relevant increase in methylation in the tumor versus normal at genomic location from cluster 4 (Mann-Whitney U p-value <2.2e^−16^).

The relevance of increases DNA methylation at MBD2 binding sites for the etiology of primary breast cancer remains to be elucidated. Our findings indicate that MBD2 is likely to bind to the MBD2 sites locations in primary cancer and may contribute to the regulation of transcription of the set of genes.

## Concluding Remarks

Taken together our data suggest that MBD2 binds primarily at highly methylated regions, with a strong preference for CpG islands overlapping promoters. A small subset of promoters bound by MBD2 at a position downstream of the transcription start site (within 1 kb) is enriched for active marks and Pol2, and that these genes are expressed at low levels. We show that binding of MBD2 alters the Pol2 distribution: increase of Pol2 (phosphorylation independent), reduction of P-Ser5 at the region immediately downstream of the promoter and decrease of P-Ser2 in the remainder of the gene body. Our analysis also highlights that MBD2 binding sites display increased methylation in primary breast cancer tissues as compared to normal samples or mammary epithelial cells. This suggests that MBD2 may bind at these loci in breast cancer instigated by increased methylation. Analysis of MBD2 binding in cancer cells will be needed to determine its consequence on transcription and contribution to the etiology or maintenance of breast cancer.

## Materials and Methods

### Cloning and stable cell lines

The pTTE retroviral vector was obtained as previously described [38].

Full length MBD2 was PCR amplified from human MBD2 plasmid (image clone collection) and then cloned using EcoR1 and Xhol site to create pTTE-MBD2.

MCF-7 cells where transfected with the pTTE-MBD2 construct and single clones where selected with 1 µg/ml of puromycin. Cells were maintained in Dulbecco's modified Eagle's medium supplemented with 10% fetal calf serum, 100 µl/ml Penicillin and 100 units/ml Streptomycin and 1 µg/ml puromycin at 37°C in 5% CO_2_.

### Antibodies and western blot

Rabbit anti-MBD2 Antibody, Bethyl Laboratories, (A301-632), GAPDH (ab8245-100) and BB2 (against TY1 epitope) were used for Western Blot analysis. The same MBD2 and BB2 together with H2A.Zac (acetyl K4+K7+K11, ab18262, sheep polyclonal, 659355) from Abcam, Pol2 (8WG16 Monoclonal Antibody, Catalog Number MMS-126R, Covance), H3K4me3 from Diagenode (pAb-MEHAHS-024, rabbit polyclonal, HC-0010), Ser5 Pol2 (phospho-CTD Ser-5, clone 3E8, Millipore Cat 04-1572) and Ser2 (phospho CTD Ser-2, clone 3E10, Millipore Cat 04-1571), N-term Pol2 (Pol II N-20 sc-899X SantaCruz) were used for ChIP-seq, Western blot analysis was performed according to standard procedure on whole cell lysates.

### Nuclear protein extraction and mass spec analysis

Nuclear extracts from TTE-MBD2 and WT MCF-7 were prepared as previously described [39]. For the immunoprecipitation 500 µg of nuclear proteins were incubated over night at 4°C with 50 µl protein A/G plus-agarose beads and 5 µg BB2 antibody, each sample was prepared in triplicates. After washes samples were subjected to on-bead trypsin digestion as previously described [40] and prepared for mass spec analysis. Peptides were eluted from stage-tips with 2×20 ul of 60% acetonitrile/0.1% formic acid. Acetonitrile was evaporated using a speed vacuum centrifuge and the sample volume was brought to 15 ul adding 0.1% formic acid. 5 ul of peptide solution was loaded on a fused silica column (75 um inner diameter, 30 cm length) (Next Advanced) packed in-house with ReproSil-Pur C18-AQ 1.8 µm resin (Dr. Maisch) using a EASY-nLC II (Thermo Scientific). Peptides were eluted over 120 minutes using a segmented gradient with increasing concentration of acetonitrile. The column was kept at a temperature of 50°C using a column oven (Sonation). Eluting peptides were sprayed directly into a QExactive mass spectrometer (Thermo Scientific). The mass spectrometer was operated in data dependent TOP10 sequencing mode. Target values for full MS scans were set to 3.000.000 and for MS/MS to 100.000 with maximum fill times of 20 ms and 120 ms, respectively. MS spectra were recorded at a resolution (m/z = 400) of 70.000 and MS/MS spectra at a resolution of 17.500. Peptides were fragmented using higher energy collision induced dissociation (HCD) with NCE = 25. The isolation window was set to 3 Th. Singly charged peaks or peaks with charge state were excluded for sequencing. Dynamic exclusion was activated and the window was set to 20 s. Data acquisition was performed using Xcalibur software. Data analysis was performed basically as described in [26] using the MaxQuant software package [41]. Statistical analysis was performed using Perseus and the interaction data was visualized using R.

### Double step-crosslinking and chromatin-immunoprecipitation

Tagged-MBD2 ChIPs were performed with MBD2 Ab (A301-632) and BB2 (against Ty1) following a double step crosslinking method [42].

Cells were trypsinized and re-suspended with PBS to a final concentration of 8×10^6^ cells/500 µl PBS. Cell suspensions were crosslinked with 1.5 mM DSG (disuccinimidyl glutarate, Thermo, #20593) for 45′ at room temperature with gentle rotation. After two washes with 500 µl PBS, cell pellets were re-suspended in 1 ml PBS and 1% formaldehyde was added for 10′ at room temperature.

Cross-linking was quenched with 125 mM glycine and after two times ice-cold PBS washes, pellets were resuspended in 270 µl lysis buffer (50 mM Tris pH 8.0, 1%SDS, 10 mMEDTA protease inhibitor) and incubated 5′ on ice.

Sonication was performed for 15′–20′ with Bioruptor sonicator (NGS, Diagenode) and lysates were centrifuged at 13000 rpm 4°C, for 5 min.

20 µl of Dynabeads protein A/G (Life Technologies) and 50 µl of Dynabeads protein G were pre-incubated for 1 h respectively with 4 µl MBD2 and 5 µg of BB2, in 1 ml IP buffer (0.01%SDS,1.1% TritonX100, 1.2 mM EDTA, 16.7 mM Tris pH 8.0, 167 mM NaCl) by gentle rocking at 4°C.

After 1 ml wash with IP buffer, antibody-loaded beads were incubated with 100 µl chromatin, 100 µl of 50 mg/ml BSA in IP buffer, 800 µl IP buffer and 1.25 µl 10 mg/ml yeast tRNA (Ambion #AM7119) over-night at 4°C.

Beads were washed subsequently with 5 different buffers: one time with IP buffers, two times with RIPA buffer (25 mM TrisHCl pH 7.6, 150 mM NaCl, 1% NP-40, 1% sodium deoxycholate, 0.1% SDS), two times with RIPA high salt buffer (1∶10 mixture with 5 M NaCl), one time with LiCl wash buffer (2 mM EDTA, 20 mM Tris pH 8, 250 mM LiCl, 1% NP-40, 1% sodium deoxycholate) and two times with TE buffer.

Beads were resuspended in 50 µl freshly prepared elution buffer (1%SDS, 0.1 M NaHCO3) supplemented with 5 mM DTT and incubated for 45′ at 65°C in a thermomixer.

Supernatants were collected and beads were re-suspended once more with 50 µl fresh elution buffer, the two supernatants were joined, supplemented with 300 mM NaCl 0.5 µl RNase cocktail and de-crosslinked for 4 hours at 65°C, shaking.

After addition of 2 µl 1 M Tris pH 6.8 and 2 µl 20 mg/mL proteinase K samples were incubated at 65°C for 1 more hour.

DNA was purified with QIAGEN columns, and 2–5 µg were used for library preparation and sequencing. 50 µl from TTE-MBD2 chromatin was also de-crosslinked and prepared for sequencing (referred as input chromatin).

For all the other chromatin-immunoprecipitations cells were fixed for 10 minutes at room temperature by the addition of formaldehyde to a final concentration of 1%, after which glycine was added to a concentration of 100 mM. Cells were then washed twice with PBS and collected into lysis buffer (150 mM NaCl, 20 mM Tris pH 8.0, 2 mM EDTA, 1% triton X-100, protease inhibitor [complete EDTA free, Roche, 04 693 132 001], 100 mM PMSF). The lysate was sonicated to an average of 300–500 bp fragments. The resulting sonicate was centrifuged at 4000×g for 5 minutes, an aliquot of 10% retained for input and the remaining material transferred to a fresh tube. 20 µl protein G or protein A/G magnetic beads were pre-incubated with the different antibodies for 4 hours and after washing away the excess of antibody, chromatin was added O.N at 4°C.

Afterwards, the complexes were washed, then reverse crosslinked for a minimum of 4 hours at 65°C. Recovered DNA was then purified using a Qiaquick spin column and eluted in 50 of 10 mM Tris pH 8.0.

Strand specific RNA (ssRNA) preparation was performed as previously described [43].

### Illumina high-throughput sequencing and data analysis

Libraries were prepared according to manufacturer's standards. Briefly 5–10 ng DNA was subjected to end repair using Klenow DNA polymerase, T4 ligase and T4 polynucleotide kinase (T4 PNK). A 3′ protruding A base was generated using Taq polymerase and Illumina adapters (GAIIX) or NEXTflex adapters (HiSeq2000) were ligated. The DNA was loaded on E-gel for size selection (300 bp), further amplified by PCR and used for cluster generation on the Illumina GAIIx or HiSeq2000 genome analyzer. The 36 (GAIIx) or 43 bp tags (HiSeq2000) were mapped to the reference human genome hg19 (NCBI build 37), using the BWA allowing one mismatch. Only uniquely mapped-reads were used for data analysis and visualization.

Peak-calling was performed with MACS 2.0 tool against a reference input sample from the same cell line (TTE-MBD2). Genomic distribution of peaks or random regions was performed with a script that annotates binding sites according to all RefSeq genes, taking into account 4 functional categories: promoters (1 kb up or downstream the TSS), exons, introns and intergenic. The random set consists of an equal number of sites having on average same length of the peaks. Each category is subsequently grouped in “CGI” or “non-CGI” according to the overlap with CpG islands. The assignment is non-overlapping, peaks are only assigned to a category once and the above order is hierarchical. Whole genome bisulfite sequencing data were analyzed as previously described [29].

Cytosine methylation levels within CpG context are present in the MCF-7_CpG_methcounts.bed.gz file and are used to calculate methylation levels (between 0 = 0% methylation and 1 = 100% methylation) and mCpG density per 1 kb genomic window.

Heatmaps and k-mean clustering together with relative average profiles (Fig 4B) were performed using a Python package available at http://simonvh.github.io/fluff/. ChIP-seq datasets used for generating average profiles and comparisons between WT and TTE-MBD2 were normalized for total number of uniquely mapped-reads. All average profiles are obtained counting tags (or methylated CpG) per 100 bp windows.

For gene expression analysis RPKM values were calculated from RNA-seq data and only expressed genes were taken into account. For H3K36me3 levels the number of tags per bp were computed over the gene bodies on all Ref-seq annotated genes.

For Ser2-P average profiles over gene bodies downstream promoters from cluster 4 only genes longer than 4 kb were taken into account. Same criteria were followed to compute mCpG density (Fig S7–S8).

R was used to generate most of the graphs.

The data generated for this work have been deposited in NCBI's Gene Expression Omnibus and are accessible through GEO Series accession number GSE54693.

The rest of the ChIP-Seq datasets (H3K27ac; H3K36me3; P300; H3K27me3; H3K9me3) were retrieved from the ENCODE data repository site (http://genome.ucsc.edu/ENCODE/).

## Supporting Information

Figure S1 ChIP qPCR for MBD2 ChIP on WT, TTE-MBD2 and Ty1 ChIP on TTE-MBD2 at some representative binding sites. The y-axis shows recoveries expressed as % of input.
(TIF)

Figure S2 Heatmap displaying tag densities in 2 MBD2 ChIP-seq replica on TTE-MBD2, Ty1 ChIP on TTE-MBD2 (“Ty1”) and WT (“Ty1 control”) and input at TTE-MBD2 binding sites around 5 kb up and downstream the center of the peaks.
(TIF)

Figure S3 Screenshots from the genome browser with examples from each cluster.
(TIF)

Figure S4 ChIP qPCR for MBD2 ChIP on WT and TTE-MBD2 tested at 1 kb downstream of a set of loci from cluster 4.
(TIF)

Figure S5 As for Fig 5C boxplots showing H3K36me3 tag densities for all Ref-seq annotated genes sorted and divided in 3 categories according to H3K36me3 levels, compared to H3K36me3 density for genes downstream annotated promoters in cluster 4.
(TIF)

Figure S6 Average profiles of Pol2 (using the antibody against CTD, 8WG16) at promoters from cluster 4, calculated 5 kb up- and downstream the TSS for TTE-MBD2 and WT MCF-7.
(TIF)

Figure S7 Boxplots showing mCpG densities for all Ref-seq annotated genes sorted and divided in 3 categories according to mCpG density, compared to density in genes downstream promoters from cluster 4.
(TIF)

Figure S8 Average methylation levels calculated 2 kb up- and downstream the TSS and the TES, for gene bodies downstream promoters from cluster 4 (as Fig 5F), and for the “high” bin (Fig S7) with highest mCpG density.
(TIF)

Table S1 Complete list of all identified proteins after Ty1 immunoprecipitation on both TTE-MBD2 and WT cells followed by mass spectrometry analysis. Results from triplicate pull-downs were analyzed with MaxQuant and label-free quantitation (LFQ).
(XLSX)

## References

[1] Ariel M, Robinson E, McCarrey JR, Cedar H (1995) Gamete-specific methylation correlates with imprinting of the murine Xist gene. Nature genetics 9: 312–315. 7773295 10.1038/ng0395-312

[2] Holliday R, Pugh JE (1975) DNA modification mechanisms and gene activity during development. Science 187: 226–232. 1111098

[3] Wutz A, Smrzka OW, Schweifer N, Schellander K, Wagner EF, et al (1997) Imprinted expression of the Igf2r gene depends on an intronic CpG island. Nature 389: 745–749. 9338788 10.1038/39631

[4] Feinberg AP, Vogelstein B (1983) Hypomethylation distinguishes genes of some human cancers from their normal counterparts. Nature 301: 89–92. 6185846 10.1038/301089a0

[5] Robertson KD, Jones PA (2000) DNA methylation: past, present and future directions. Carcinogenesis 21: 461–467. 10688866 10.1093/carcin/21.3.461

[6] Bird AP (1986) CpG-rich islands and the function of DNA methylation. Nature 321: 209–213. 2423876 10.1038/321209a0

[7] Mohn F, Weber M, Rebhan M, Roloff TC, Richter J, et al (2008) Lineage-specific polycomb targets and de novo DNA methylation define restriction and potential of neuronal progenitors. Molecular cell 30: 755–766. 18514006 10.1016/j.molcel.2008.05.007

[8] Jones PA, Baylin SB (2002) The fundamental role of epigenetic events in cancer. Nature reviews Genetics 3: 415–428. 10.1038/nrg816 12042769

[9] Deaton AM, Bird A (2011) CpG islands and the regulation of transcription. Genes & development 25: 1010–1022. 21576262 10.1101/gad.2037511 PMC3093116

[10] Hellman A, Chess A (2007) Gene body-specific methylation on the active X chromosome. Science 315: 1141–1143. 17322062 10.1126/science.1136352

[11] Wolf SF, Jolly DJ, Lunnen KD, Friedmann T, Migeon BR (1984) Methylation of the hypoxanthine phosphoribosyltransferase locus on the human X chromosome: implications for X-chromosome inactivation. Proceedings of the National Academy of Sciences of the United States of America 81: 2806–2810. 6585829 10.1073/pnas.81.9.2806 PMC345159

[12] Ball MP, Li JB, Gao Y, Lee JH, LeProust EM, et al (2009) Targeted and genome-scale strategies reveal gene-body methylation signatures in human cells. Nature biotechnology 27: 361–368. 10.1038/nbt.1533 PMC3566772 19329998

[13] Klose RJ, Bird AP (2006) Genomic DNA methylation: the mark and its mediators. Trends in biochemical sciences 31: 89–97. 16403636 10.1016/j.tibs.2005.12.008

[14] Fournier A, Sasai N, Nakao M, Defossez PA (2012) The role of methyl-binding proteins in chromatin organization and epigenome maintenance. Briefings in functional genomics 11: 251–264. 22184333 10.1093/bfgp/elr040

[15] Laget S, Joulie M, Le Masson F, Sasai N, Christians E, et al (2010) The human proteins MBD5 and MBD6 associate with heterochromatin but they do not bind methylated DNA. PloS one 5: e11982. 20700456 10.1371/journal.pone.0011982 PMC2917364

[16] Barr H, Hermann A, Berger J, Tsai HH, Adie K, et al (2007) Mbd2 contributes to DNA methylation-directed repression of the Xist gene. Molecular and cellular biology 27: 3750–3757. 17353271 10.1128/MCB.02204-06 PMC1900000

[17] Zhang Y, Ng HH, Erdjument-Bromage H, Tempst P, Bird A, et al (1999) Analysis of the NuRD subunits reveals a histone deacetylase core complex and a connection with DNA methylation. Genes & development 13: 1924–1935. 10444591 10.1101/gad.13.15.1924 PMC316920

[18] Zhu D, Hunter SB, Vertino PM, Van Meir EG (2011) Overexpression of MBD2 in glioblastoma maintains epigenetic silencing and inhibits the antiangiogenic function of the tumor suppressor gene BAI1. Cancer research 71: 5859–5870. 21724586 10.1158/0008-5472.CAN-11-1157 PMC3165103

[19] Martin V, Jorgensen HF, Chaubert AS, Berger J, Barr H, et al (2008) MBD2-mediated transcriptional repression of the p14ARF tumor suppressor gene in human colon cancer cells. Pathobiology: journal of immunopathology, molecular and cellular biology 75: 281–287. 10.1159/000151708 18931530

[20] Stefanska B, Suderman M, Machnes Z, Bhattacharyya B, Hallett M, et al (2013) Transcription onset of genes critical in liver carcinogenesis is epigenetically regulated by methylated DNA-binding protein MBD2. Carcinogenesis 34: 2738–2749. 23955541 10.1093/carcin/bgt273

[21] Le Guezennec X, Vermeulen M, Brinkman AB, Hoeijmakers WA, Cohen A, et al (2006) MBD2/NuRD and MBD3/NuRD, two distinct complexes with different biochemical and functional properties. Molecular and cellular biology 26: 843–851. 16428440 10.1128/MCB.26.3.843-851.2006 PMC1347035

[22] Hendrich B, Bird A (1998) Identification and characterization of a family of mammalian methyl-CpG binding proteins. Molecular and cellular biology 18: 6538–6547. 9774669 10.1128/mcb.18.11.6538 PMC109239

[23] Gunther K, Rust M, Leers J, Boettger T, Scharfe M, et al (2013) Differential roles for MBD2 and MBD3 at methylated CpG islands, active promoters and binding to exon sequences. Nucleic acids research 41: 3010–3021. 23361464 10.1093/nar/gkt035 PMC3597697

[24] Baubec T, Ivanek R, Lienert F, Schubeler D (2013) Methylation-dependent and -independent genomic targeting principles of the MBD protein family. Cell 153: 480–492. 23582333 10.1016/j.cell.2013.03.011

[25] Costessi A, Mahrour N, Sharma V, Stunnenberg R, Stoel MA, et al (2012) The human EKC/KEOPS complex is recruited to Cullin2 ubiquitin ligases by the human tumour antigen PRAME. PLoS One 7: e42822. 22912744 10.1371/journal.pone.0042822 PMC3418287

[26] Hubner NC, Bird AW, Cox J, Splettstoesser B, Bandilla P, et al (2010) Quantitative proteomics combined with BAC TransgeneOmics reveals in vivo protein interactions. The Journal of cell biology 189: 739–754. 20479470 10.1083/jcb.200911091 PMC2872919

[27] Tian B, Yang J, Brasier AR (2012) Two-step cross-linking for analysis of protein-chromatin interactions. Methods in molecular biology 809: 105–120. 22113271 10.1007/978-1-61779-376-9_7 PMC4148016

[28] Feng J, Liu T, Qin B, Zhang Y, Liu XS (2012) Identifying ChIP-seq enrichment using MACS. Nature protocols 7: 1728–1740. 22936215 10.1038/nprot.2012.101 PMC3868217

[29] Habibi E, Brinkman AB, Arand J, Kroeze LI, Kerstens HH, et al (2013) Whole-genome bisulfite sequencing of two distinct interconvertible DNA methylomes of mouse embryonic stem cells. Cell stem cell 13: 360–369. 23850244 10.1016/j.stem.2013.06.002

[30] Vakoc CR, Mandat SA, Olenchock BA, Blobel GA (2005) Histone H3 lysine 9 methylation and HP1gamma are associated with transcription elongation through mammalian chromatin. Molecular cell 19: 381–391. 16061184 10.1016/j.molcel.2005.06.011

[31] Brinkman AB, Roelofsen T, Pennings SW, Martens JH, Jenuwein T, et al (2006) Histone modification patterns associated with the human X chromosome. EMBO reports 7: 628–634. 16648823 10.1038/sj.embor.7400686 PMC1479594

[32] Baubec T, Ivánek R, Lienert F, Schübeler D (2013) Methylation-Dependent and -Independent Genomic Targeting Principles of the MBD Protein Family. Cell 153: 480–492. 23582333 10.1016/j.cell.2013.03.011

[33] Komarnitsky P, Cho EJ, Buratowski S (2000) Different phosphorylated forms of RNA polymerase II and associated mRNA processing factors during transcription. Genes Dev 14: 2452–2460. 11018013 10.1101/gad.824700 PMC316976

[34] Jonkers I, Kwak H, Lis JT (2014) Genome-wide dynamics of Pol II elongation and its interplay with promoter proximal pausing, chromatin, and exons. eLife. 10.7554/eLife.02407 PMC4001325 24843027

[35] Veloso A, Kirkconnell KS, Magnuson B, Biewen B, Paulsen MT, et al (2014) Rate of elongation by RNA polymerase II is associated with specific gene features and epigenetic modifications. Genome Res. 10.1101/gr.171405.113 PMC4032854 24714810

[36] Hon GC, Hawkins RD, Caballero OL, Lo C, Lister R, et al (2012) Global DNA hypomethylation coupled to repressive chromatin domain formation and gene silencing in breast cancer. Genome Res 22: 246–258. 22156296 10.1101/gr.125872.111 PMC3266032

[37] Ruike Y, Imanaka Y, Sato F, Shimizu K, Tsujimoto G (2010) Genome-wide analysis of aberrant methylation in human breast cancer cells using methyl-DNA immunoprecipitation combined with high-throughput sequencing. BMC Genomics 11: 137. 20181289 10.1186/1471-2164-11-137 PMC2838848

[38] Costessi A, Mahrour N, Tijchon E, Stunnenberg R, Stoel MA, et al (2011) The tumour antigen PRAME is a subunit of a Cul2 ubiquitin ligase and associates with active NFY promoters. The EMBO journal 30: 3786–3798. 21822215 10.1038/emboj.2011.262 PMC3173790

[39] Smits AH, Jansen PW, Poser I, Hyman AA, Vermeulen M (2013) Stoichiometry of chromatin-associated protein complexes revealed by label-free quantitative mass spectrometry-based proteomics. Nucleic acids research 41: e28. 23066101 10.1093/nar/gks941 PMC3592467

[40] Hubner NC, Mann M (2011) Extracting gene function from protein-protein interactions using Quantitative BAC InteraCtomics (QUBIC). Methods 53: 453–459. 21184827 10.1016/j.ymeth.2010.12.016

[41] Cox J, Mann M (2008) MaxQuant enables high peptide identification rates, individualized p.p.b.-range mass accuracies and proteome-wide protein quantification. Nature biotechnology 26: 1367–1372. 10.1038/nbt.1511 19029910

[42] Shimbo T, Du Y, Grimm SA, Dhasarathy A, Mav D, et al (2013) MBD3 Localizes at Promoters, Gene Bodies and Enhancers of Active Genes. PLoS genetics 9: e1004028. 24385926 10.1371/journal.pgen.1004028 PMC3873231

[43] Ovaska K, Matarese F, Grote K, Charapitsa I, Cervera A, et al (2013) Integrative analysis of deep sequencing data identifies estrogen receptor early response genes and links ATAD3B to poor survival in breast cancer. PLoS computational biology 9: e1003100. 23818839 10.1371/journal.pcbi.1003100 PMC3688481

