# Supplementary figures and images for: Genome-Wide Binding of MBD2 Reveals Strong Preference for Highly Methylated Loci
Source: PLoS One. 2014 Jun 13;9(6):e99603. doi: 10.1371/journal.pone.0099603 (PMC4057170; doi:10.1371/journal.pone.0099603)

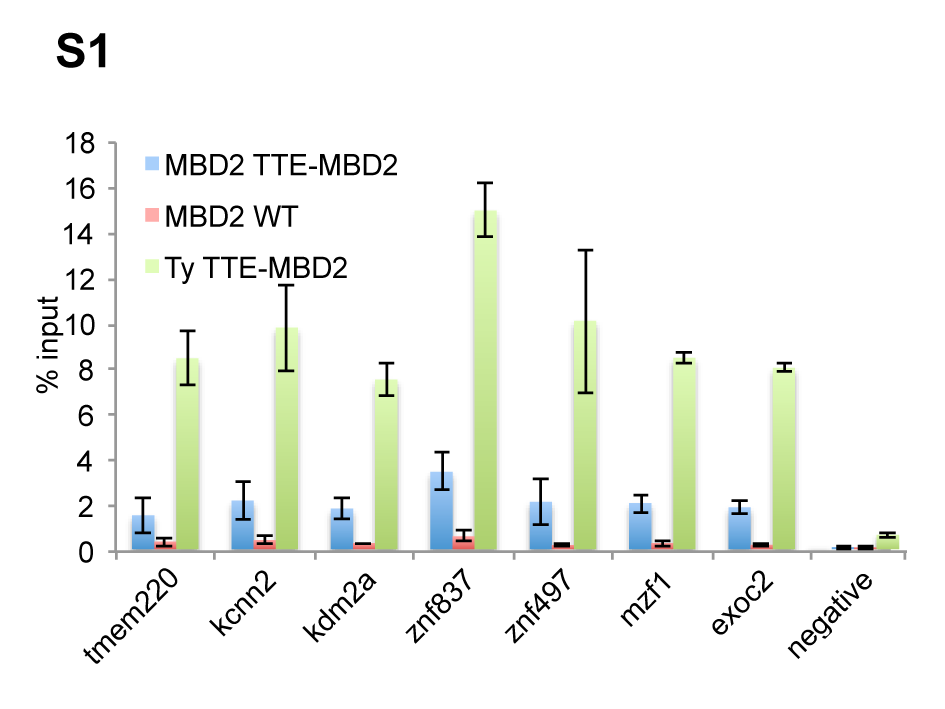

Supplement: Figure S1 — ChIP qPCR for MBD2 ChIP on WT, TTE-MBD2 and Ty1 ChIP on TTE-MBD2 at some representative binding sites. The y-axis shows recoveries expressed as % of input. (TIF) [file pone.0099603.s001.tif]

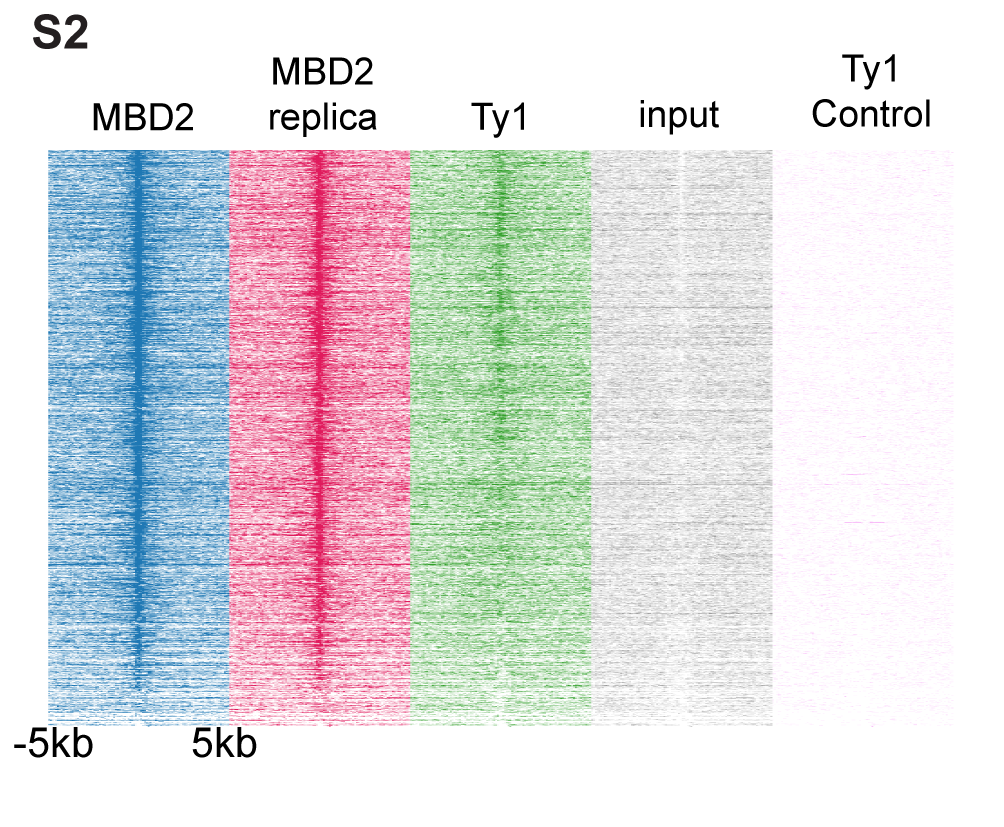

Supplement: Figure S2 — Heatmap displaying tag densities in 2 MBD2 ChIP-seq replica on TTE-MBD2, Ty1 ChIP on TTE-MBD2 (“Ty1”) and WT (“Ty1 control”) and input at TTE-MBD2 binding sites around 5 kb up and downstream the center of the peaks. (TIF) [file pone.0099603.s002.tif]

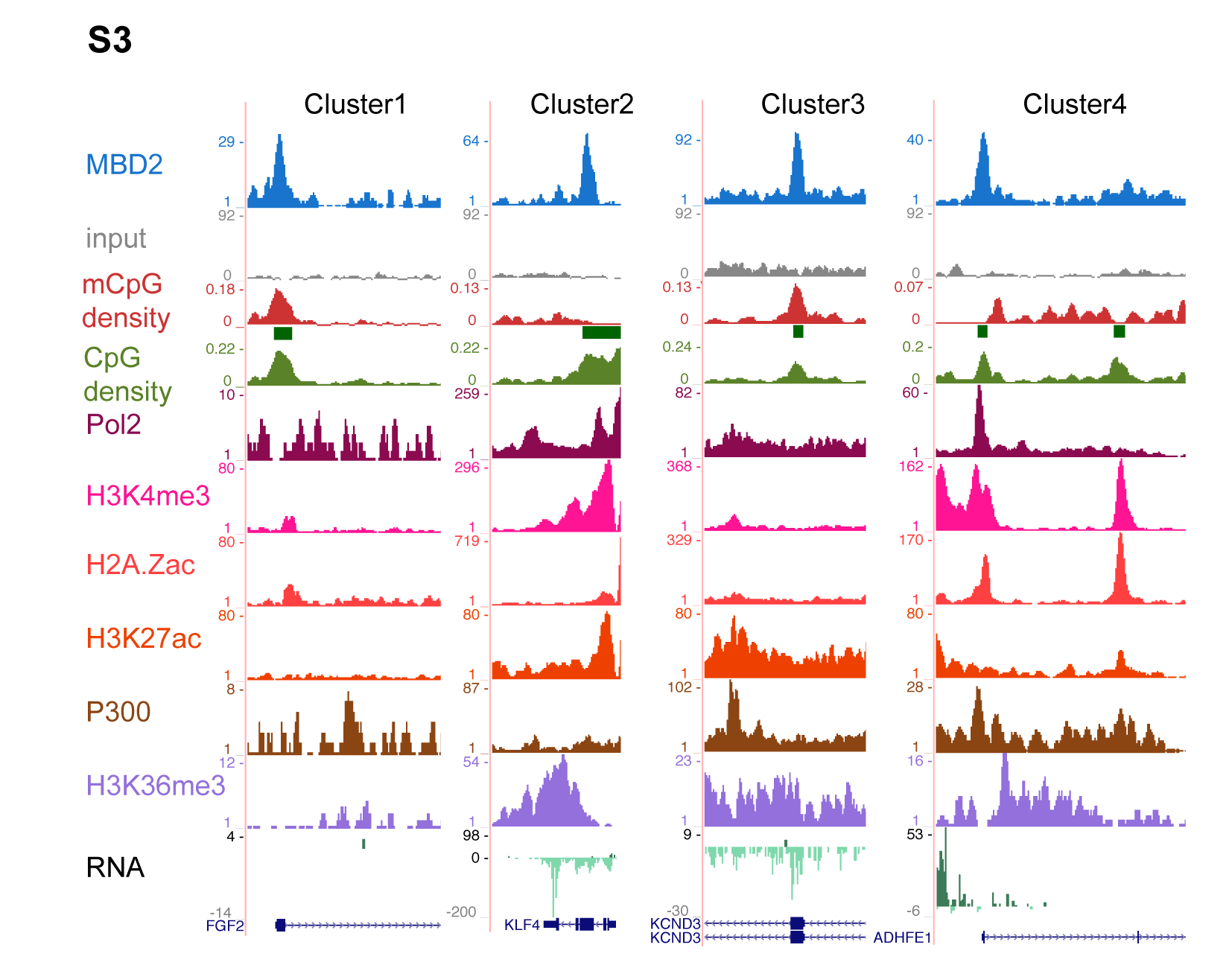

Supplement: Figure S3 — Screenshots from the genome browser with examples from each cluster. (TIF) [file pone.0099603.s003.tif]

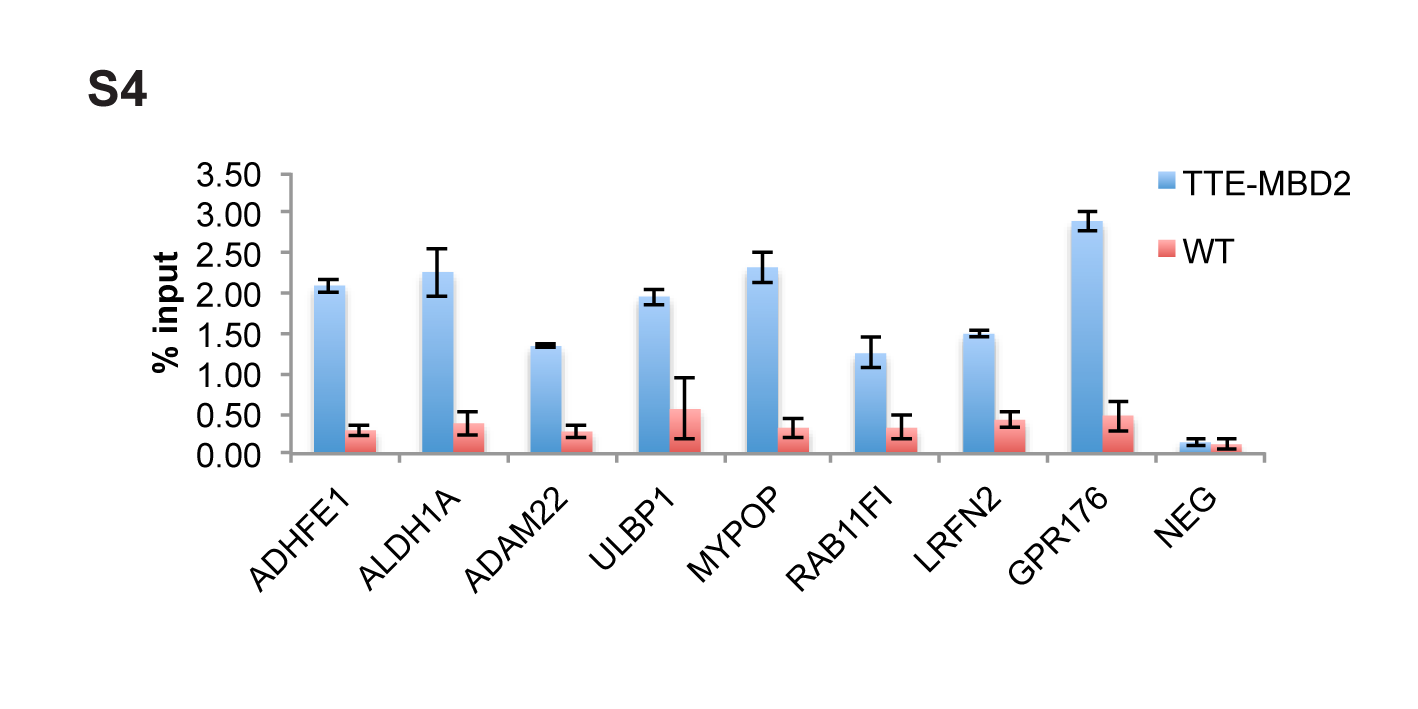

Supplement: Figure S4 — ChIP qPCR for MBD2 ChIP on WT and TTE-MBD2 tested at 1 kb downstream of a set of loci from cluster 4. (TIF) [file pone.0099603.s004.tif]

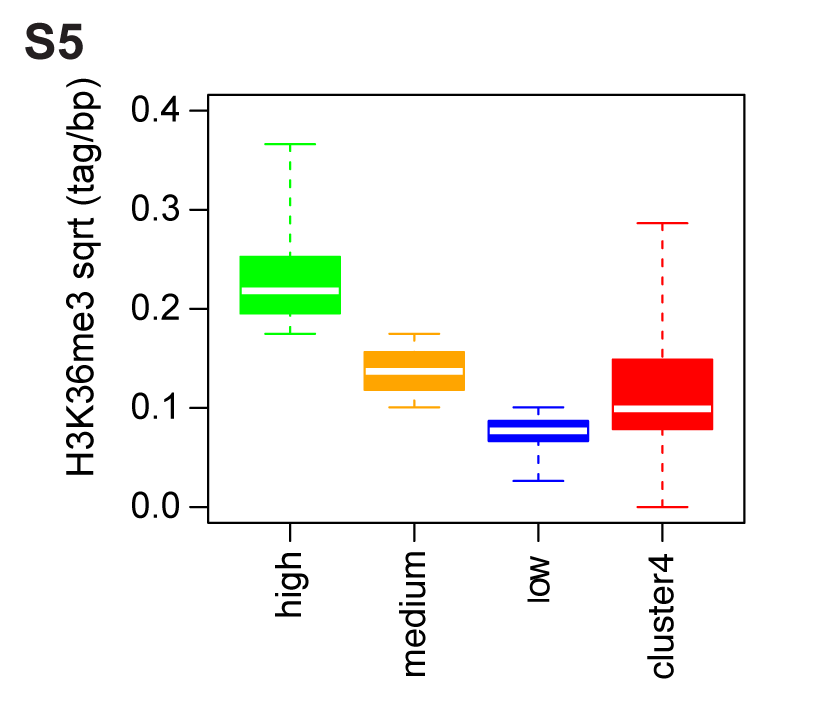

Supplement: Figure S5 — As for Fig 5C boxplots showing H3K36me3 tag densities for all Ref-seq annotated genes sorted and divided in 3 categories according to H3K36me3 levels, compared to H3K36me3 density for genes downstream annotated promoters in cluster 4. (TIF) [file pone.0099603.s005.tif]

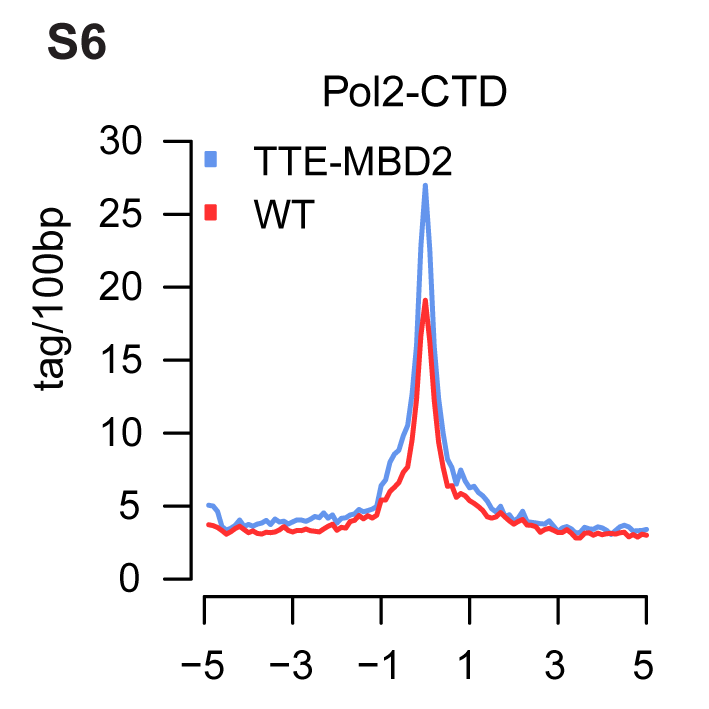

Supplement: Figure S6 — Average profiles of Pol2 (using the antibody against CTD, 8WG16) at promoters from cluster 4, calculated 5 kb up- and downstream the TSS for TTE-MBD2 and WT MCF-7. (TIF) [file pone.0099603.s006.tif]

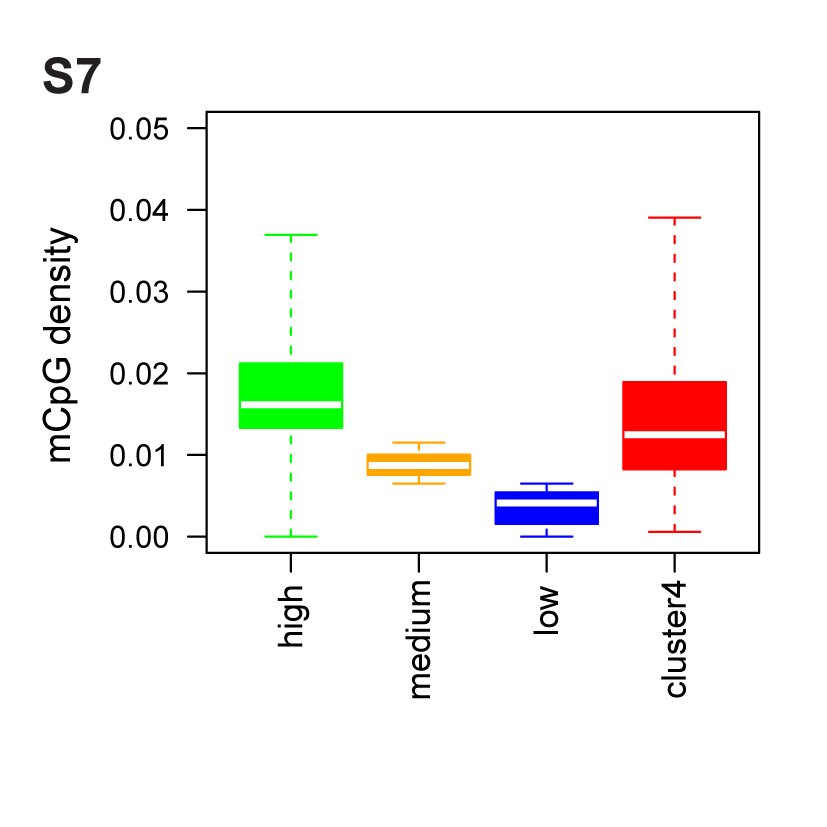

Supplement: Figure S7 — Boxplots showing mCpG densities for all Ref-seq annotated genes sorted and divided in 3 categories according to mCpG density, compared to density in genes downstream promoters from cluster 4. (TIF) [file pone.0099603.s007.tif]

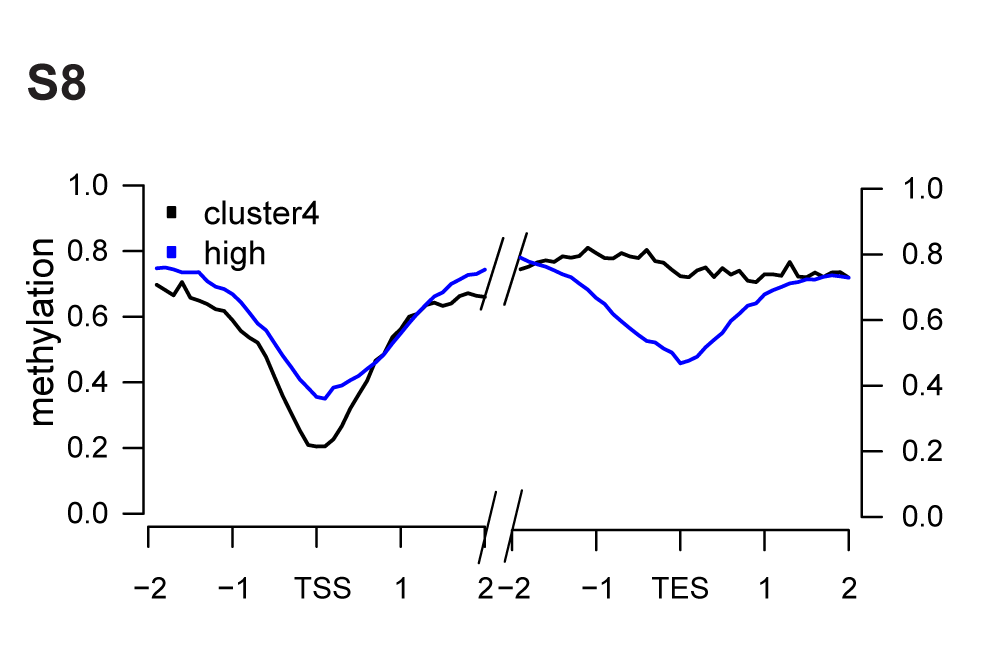

Supplement: Figure S8 — Average methylation levels calculated 2 kb up- and downstream the TSS and the TES, for gene bodies downstream promoters from cluster 4 (as Fig 5F), and for the “high” bin (Fig S7) with highest mCpG density. (TIF) [file pone.0099603.s008.tif]
